# BRCA1 and CtIP Are Both Required to Recruit Dna2 at Double-Strand Breaks in Homologous Recombination

**DOI:** 10.1371/journal.pone.0124495

**Published:** 2015-04-24

**Authors:** Nguyen Ngoc Hoa, Junya Kobayashi, Masato Omura, Mayumi Hirakawa, Soo-Hyun Yang, Kenshi Komatsu, Tanya T. Paull, Shunichi Takeda, Hiroyuki Sasanuma

**Affiliations:** 1 Department of Radiation Genetics, Graduate School of Medicine, Kyoto University, Yoshidakonoe, Sakyo-ku, Kyoto, Japan; 2 Department of Genome Repair Dynamics, Radiation Biology Center, Kyoto University, Yoshidakonoe, Sakyo-ku, Kyoto, Japan; 3 The Howard Hughes Medical Institute, The Department of Molecular Biosciences, and The Institute for Cellular and Molecular Biology, The University of Texas at Austin, Austin, United States of America; Dana-Farber/Harvard Cancer Institute, UNITED STATES

## Abstract

Homologous recombination plays a key role in the repair of double-strand breaks (DSBs), and thereby significantly contributes to cellular tolerance to radiotherapy and some chemotherapy. DSB repair by homologous recombination is initiated by 5’ to 3’ strand resection (DSB resection), with nucleases generating the 3’ single-strand DNA (3’ssDNA) at DSB sites. Genetic studies of *Saccharomyces cerevisiae* demonstrate a two-step DSB resection, wherein CtIP and Mre11 nucleases carry out short-range DSB resection followed by long-range DSB resection done by Dna2 and Exo1 nucleases. Recent studies indicate that CtIP contributes to DSB resection through its non-catalytic role but not as a nuclease. However, it remains elusive how CtIP contributes to DSB resection. To explore the non-catalytic role, we examined the dynamics of Dna2 by developing an immuno-cytochemical method to detect ionizing-radiation (IR)-induced Dna2-subnuclear-focus formation at DSB sites in chicken DT40 and human cell lines. Ionizing-radiation induced Dna2 foci only in *wild-type* cells, but not in Dna2 depleted cells, with the number of foci reaching its maximum at 30 minutes and being hardly detectable at 120 minutes after IR. Induced foci were detectable in cells in the G2 phase but not in the G1 phase. These observations suggest that Dna2 foci represent the recruitment of Dna2 to DSB sites for DSB resection. Importantly, the depletion of CtIP inhibited the recruitment of Dna2 to DSB sites in both human cells and chicken DT40 cells. Likewise, a defect in breast cancer 1 (BRCA1), which physically interacts with CtIP and contributes to DSB resection, also inhibited the recruitment of Dna2. Moreover, CtIP physically associates with Dna2, and the association is enhanced by IR. We conclude that BRCA1 and CtIP contribute to DSB resection by recruiting Dna2 to damage sites, thus ensuring the robust DSB resection necessary for efficient homologous recombination.

## Introduction

DSBs are the most dangerous type of DNA damage, as a single unrepaired DSB can trigger apoptosis. DSBs are generated during physiological replication and can be induced by chemotherapeutic agents such as camptothecin, cisplatin and poly[ADP ribose]polymerase (PARP) inhibitors, as well as by IR. DSBs are repaired by the two major pathways: homologous recombination (HR) and non-homologous end-joining (NHEJ). HR, but not NHEJ, repairs DSBs induced by chemotherapeutic agents as well as those that occur during physiological replication [1, 2]. HR can also repair IR-induced DSBs in the S and G_2_ phases.

HR is carried out in a series of steps, beginning with DSB resection, specifically the 5’ to 3’ strand resection of DSBs [3, 4]. The resulting 3’-overhang is coated with a single-strand binding protein known as replication protein A (RPA). RPA is subsequently replaced by polymerized Rad51 recombinase, which results in the formation of subnuclear Rad51 foci. Polymerized Rad51 then performs homology search and strand invasion into the intact homologous sequences. In *Saccharomyces cerevisiae*, DSB resection is carried out by four nucleases: Mre11-Rad50-Xrs2 (the MRX complex), Sae2, Dna2 and Exo1 [5–9]. The MRX complex and Sae2 are the orthologs of human Mre11-Rad50-Nbs1 (the MRN complex) and CtIP, respectively [10, 11]. MRX and Sae2 cooperatively initiate HR by removing up to a few hundred nucleotides from the 5’ end of DSBs, resulting in short-range resection [5, 6]. Dna2 and Exo1 then perform the further long-range resection of two or more kilobases.

Recent studies have suggested that yeast Sae2 and mammalian CtIP contribute to DSB resection in a different manner. Unlike Sae2, CtIP plays the central role in DSB resection, as inactivation of CtIP as well as Mre11 is mortal to cells [12, 13], and CtIP depletion severely diminishes both the formation of Rad51 foci and the efficiency of HR [11, 12, 14]. More importantly, although the nuclease activity of Sae2 is required for DSB resection, CtIP facilitates DSB resection through its non-catalytic functioning while contributes to cellular tolerance to topoisomerase poisons as a nuclease[15, 16]. It remains elusive how CtIP facilitates DSB resection. Another unsolved question is whether or not BRCA1 contributes to DSB resection. The role of BRCA1 in DSB resection is supported by the following: (i) BRCA1 physically associates with CtIP [17, 18], and (ii) the severe defect in the HR of *BRCA1* ^*-/-*^ cells is restored to near normal by the additional inactivation of 53BP1, a protein involved in NHEJ [19, 20].

We found that the inactivation of nuclease activity associated with Dna2 completely inhibited DSB resection, as did the depletion of CtIP in both human cells and chicken DT40 cells [11, 21]. Depletion of CtIP significantly suppressed Dna2-focus formation at IR-induced DSB sites. These observations suggest a previously unappreciated interdependency between CtIP and Dna2, where CtIP recruits Dna2 to DSB sites and the nuclease activity of Dna2 is responsible for DSB resection. *BRCA1* ^*-/-*^ cells also displayed a defect in Dna2-focus formation whereas *BRCA1* ^*-/-*^ */53BP1* ^*-/-*^ cells displayed nearly normal Dna2 foci. We propose that 53BP1 and BRCA1 may control the CtIP—dependent recruitment of Dna2 to DNA damage sites for subsequent DSB resection.

## Materials and Methods

### Cell lines and Culture Conditions

The DT40 cell line is derived from chicken B lymphoma [22] and was cultured at 39.5°C with 5% CO_2_ in RMPI-1640 medium (Nacalai Tesque, Kyoto, Japan) with glutamine (11875, Invitrogen, US) supplemented with 1% chicken serum (GIBCO-BRL, Grand Island, NY, USA), 10% heated-inactivated fetal bovine serum (FBS) (100–106, Gemini Bio-Products, West Sacramento, CA), 50 μM mercaptoethanol (Invitrogen), 50 U/ml penicillin and 50 μg/ml streptomycin (Nacalai Tesque). Doxycycline was added at a final concentration of 100 ng/ml to inactivate the expression of the tetracyclin repressible promoter.

Human cell lines (obtained from American Type Culture Collection, ATCC) were maintained at 37°C with 5% CO_2_ in DMEM media (Nacalai Tesque) supplemented with 10% FBS (Gemini Bio-Products) for U2OS and MCF7 cell lines or in RPMI-1640 (Nacalai Tesque) with 10% FBS (Gemini Bio-Products) for HCC1937 cell lines.

### Plasmid Constructs and Transfection

All primers used for plasmid construction are shown in Table 1. To generate a *DNA2* knockout construct, genomic DNA was amplified with the following primers: F1 and R1 (2.9 kb) for the left arm and F2 and R2 (2.7 kb) for the right arm. Each product cloned was combined on pBLUESCRIPT-SK (pBS-SK) plasmid, after which the histidinol dehydrogenase (hisD) gene was introduced with *Bam*HI. To generate the D245A (aspartic acid replaced by alanine at position 245) knockin construct, the mutation was introduced by polymerase chain reaction (PCR) using two sets of primers: F1, F3, R3, and R1. The knockout and knockin constructs were digested with *Xho*I for linearization. Linearized plasmid (30 μg) was transfected into 10^7^ cells. The genomic DNA was digested with *Stu*I and *Bam*HI for Southern blotting. 0.6 kb DNA fragment amplified with F4 and R4 from genomic DNA was used as probes for Southern blotting. The expression of *DNA2* ^*-/D245A*^ transcript was confirmed by RT-PCR using F5 and R5. Each amplicon, 430 bp for D245A fragment, was digested using *Bst*UI. To construct a plasmid for the inducible expression of *DNA2*, c*DNA2* was cloned downstream from the tetracycline promoter with *Sal*I and *Bam*HI. Transfection for chicken DT40 cells was performed as previously described [12].

**Table 1:** Primer list used for generation of knockout and knockin DNA2 and CtIP mutants.

Primer	Sequence
F1	GTCTAGAGAAAAGCGGTGCTGAGTGAAAGGTTTA
F2	GGGATCCACTTTGCATGTTTGAATATGAAAATGGG
F3	AAGGGAAAGATCGCGGTTACAGCCAG
F4	GAGTGTGGTTCACGGCAAACACTCGTT
F5	GAAACAAACAGAGATAATGCAGGAGA
F6	GGCGGCCGCAAAGATATCTGAACAGTTCCAGCAACTGCA
F7	GGGATCCTTGTCCTGCATGCTAAAACTAAATCTTGTG
F8	GAGGAGGAAAAAGGAGGCTAGGCATATCGCATATACGGAGCATACGCA
F9	CTAGAGTTTGAGAACATCCGACAGCAGAATCTTAAA
R1	GGGATCCAGATTACACCACATACAAACCAAATG
R2	GCTCGAGGCATCTGCAAACTCCAGTTTCAGC
R3	CTGGCTGTAACCGCGATCTTTCCCTT
R4	GGATTACGCACAAAGTCTTCTGTCCAC
R5	GTTCCAGTAACAGGATACATAGTACC
R6	GGGATCCTCATATCGTTTAAGAAAACAAGAATTTAGA
R7	TGCGTATGCTCCGTATATGCGATATGCCTAGCCTCCTTTTTCCTCCTC
R8	CCTCGAGATGAAGAAGAGATACTAGTGGCAGACACCT
R9	CTAGTATCTCTTCTTCATGATGACTGCTCG

To generate a *CtIP* knockout construct, genomic DNA was amplified with F6 and R6 for the left arm, which was cloned in pBS-SK with *Not*I and *Bam*HI. To generate the N183A,R187A knockin construct, the mutation was introduced by PCR using two sets of primers: F7, F8, R7, and R8 and the product was cloned into pBS-SK with *Bam*HI and *Xho*I. The construct was digested with *Xho*I for linearization. The expression of *CtIP* ^*N183A*,*R187A/-/-*^ transcript was amplified by RT-PCR using F9 and R9 and digested with *Bfa*I.

### Cell Counting, Cell Cycle Analysis, and Cell Synchronization

Cells were counted and cell-cycle analysis was carried out using a flow cytometer (Becton Dickinson, US). Immediately before being counted, cells were mixed with reference beads (Polyscience Ins., US) and propidium iodide. Cells were treated with BrdU (final concentration 20 μM) for 10 minutes and then fixed with 70% EtOH for the cell-cycle analysis. Cells were then incubated with FITC-conjugated anti-BrdU (1/1000, Pharmingen, US) and propidium iodide. Fluorescence data were displayed as dot plots using Cell Quest and Diva software. HeLa cells were synchronized using a double thymidine block as described previously [23]. In brief, cells were incubated with thymidine (final concentration 2 mM) for 18 hours, followed by washing with PBS and then releasing cells into fresh medium without thymidine. After incubating for 9 hours to release, cells were again incubated with 2 mM thymidine to arrest at early S- phase for 17 hours, followed by releasing into fresh medium at the time zero. Cells were harvested after 8 hour incubation to obtain G2 fraction.

### siRNA Knockdown Assay

siRNA oligos were purchased from QIAGEN (CtIP) and Dharmacom (set of 4, ON-TARGETplus, DNA2). Transfection was done using Lipofectamine 2000 (Life Technologies) according to the attached protocol. After transfection, cells were cultured in fresh media for 48 hours prior to being harvested for Western blotting and immunostaining assays.

### Immunoprecipitation and Western Blotting

Human DNA2 and CtIP were detected using anti-CtIP (1/1000, rabbit, Bethyl) and anti-DNA2 (1/1000, rabbit, Abcam). To detect protein interaction *in vivo*, cells (1 x 10^7^ cells for U2OS and ATLD2) exposed to 6 Gy γ-ray irradiation were incubated for 1 hour. Lysate was prepared from cells pretreated with paraformaldehyde (final concentration 0.05%) for 10 minutes before collection. The lysis buffer contained HEPES-KOH (ph7.5), 300mM KCl, 0.1% Triton X-100, and protease cocktail (Complete, Roche). CtIP and Dna2 were detected using anti-rabbit IgG conjugated with HRP (RPN4301, GE Healthcare).

### Chromosome Aberration Analysis

Cells were treated with 0.1 μg/ml colcemid (Invitrogen, Carlsbad, CA) for 3 hours. Cells were suspended in 75mM potassium chloride for 15 minutes, washed with Carnoy’s solution (a 3:1 mixture of methanol and acetic acid), dropped on slides, and stained with a 5% Giemsa solution for 10 minutes.

### Protein Purification and *In Vitro* Association of Human CtIP and Dna2

Human CtIP purification from insect cells: Cells were lysed in buffer A (20 mM Tris pH 8.0, 100 mM NaCl, 10% glycerol, and 1 mM DTT) supplemented with 0.5% Triton, 2.5 mM Na_4_P_2_O_7_, 1 mM Glycerol-ß-phosphate, 5 mM PMSF. Lysate was sonicated on ice until homogenous and insoluble material removed by centrifugation (100,000xg) for one hour. The supernatant was bound to 2 ml of anti-Flag M2 agarose resin slurry (Sigma) at 4°C for one hour followed by washing on a column with 20 ml buffer A, 10 ml 0.5 M LiCl, and 20 ml buffer A. The protein was eluted with 10 ml buffer A containing Flag peptide (Sigma) at a concentration of 100 μg/ml. Eluted protein was loaded onto a 1 ml Hitrap SP column (GE), washed with 20 ml buffer A and eluted with buffer A containing 0.6 M NaCl. The peak fractions were dialyzed twice against fresh 300 ml buffer A for one hour and flash frozen in liquid nitrogen. Dna2 protein was expressed in Sf21 insect cells using the baculovirus expression system. Dna2 expressing cells were lysed by homogenization and sonicated three times for 20 seconds in A buffer containing 0.5% tween-20 and 1mM PMSF (phenylmethylsulfonyl fluoride). The lysate was centrifuged for 1 hour at 35,000 rpm at 4°C. The supernatant was incubated with ~1ml M2 anti-Flag antibody-conjugated agarose resin (Sigma) with rotation at 4°C for 1 hour. After incubation the lysate with resin was centrifuged for 3 min at 1000g. After removing the supernatant, the remaining resin was washed with 20 ml of A buffer twice and was eluted with 5 ml of A buffer containing 0.8mg/ml 3X Flag peptide (Sigma). The peptide was incubated with the resin for 20 min before elution. The Flag eluent was then loaded onto 1 ml HiTrap SP column (G.E.) and washed with buffer A then eluted with buffer A containing 500 mM NaCl. The eluted protein fractions were dialyzed in A buffer and the dialyzed fractions were aliquoted, frozen in liquid nitrogen, and stored at -80°C. Purified recombinant CtIP and Dna2 were incubated on ice for 1 hour in a 100 μl reaction containing buffer A. After incubation, CtIP was immunoprecipitated using 5 μg of anti-CtIP antibody (Bethyl) and 10 μl of Protein A resin (Sigma). The beads was washed 3 times with buffer A and separated on a 6% SDS-PAGE. The gel was transferred onto a PVDF membrane, which was blotted against anti-Dna2 (Pierce: PA5-23691), and further visualized with horseradish peroxidase-conjugated anti-rabbit secondary antibody by chemiluminescence (Pierce).

### Subnuclear Focus Formation Assay

After harvest, cells were fixed by 4% paraformaldehyde for 10 minutes, followed by permeabilization using 0.5% NP-40 in PBS for 10 minutes. Fixed cells were treated with anti-Rad51 (1/1000, rabbit, Bioacademia, Japan) and FITC-conjugated anti-rabbit (1/1000, Molecular Probes, US). Rad51 foci were counted in a minimum of 100 cells were visualized at the indicated times. Dna2 (1/250, rabbit, Abcam) and γH2AX (1/1000, mouse, Millipore) foci were visualized by anti-rabbit Alexa Fluor 594 (1/1000, Life Technologies) and anti-mouse Alexa Fluor 488 (1/1000, Life Technologies), respectively, then analyzed using a BZ-9000 fluorescence microscope (Keyence, Japan). The number of Dna2 and γH2AX foci was automatically counted using BZ-Analyzer-II software (Keyence). Signals over 0.1 square μm on each nucleus were extracted and all images were normalized with the same value to cut off background signals

## Results

### Dna2 Nuclease Activity is Essential for HR

**Fig 1:**
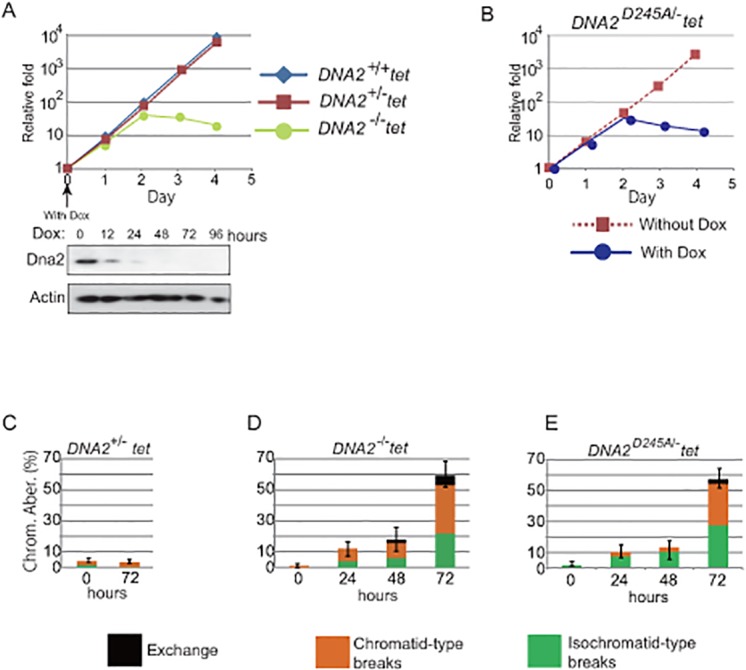
Lack of Dna2 protein causes cell-cycle arrest and increases spontaneous chromosomal aberrations. (A, B) Growth curve for *wild-type* and *DNA2* mutants. Doxycycline was added at time zero to the cell culture of the indicated genotypes (A, upper graph) and the level of Dna2 protein in the *DNA2* ^*-/-*^ */tet-DNA2* cells was analyzed at 0, 12, 24, 48, 72, and 96 hours following doxycycline treatment by western blot (A, lower panels). “With Dox” and “without Dox” in B indicate that *DNA2* ^*D245A/-*^ */tet-DNA2* cells were treated and untreated with doxycycline, respectively. (C-E) Chromosomal aberrations spontaneously arising in mitotic cells carrying the indicated genotypes after addition of doxycycline at time zero. At least 150 cells were examined, and the three indicated types of aberration were separately counted. The y-axis shows the percentage of chromosomal aberrations among the counted mitotic cells.

The chicken *DNA2* gene is located on chromosome VII (18 exons; ~14.5 kb) and encodes a 992 amino-acid protein. To explore the function of Dna2, we generated *DNA2-*gene-disruption constructs to remove seven exons encoding 282 to 630 amino acids (S1A Fig) and verified gene-targeting events using Southern blotting (S1B Fig). To conditionally disrupt the *DNA2* gene, we stably transfected DNA construct expressing the c*DNA2* gene under the control of a tetracycline-repressible promoter (tet) into the *DNA2* ^*+/-*^ cells (*DNA2* ^*+/-*^ */tet-DNA2*). We then deleted the second *DNA2* allele to generate *DNA2* ^*-/-*^ */tet-DNA2* cells. To inhibit expression of the *tet-DNA2* transgene, we added doxycycline (dox, a tetracycline analog) and confirmed no detectable Dna2 transcripts (S1C Fig) or protein after 48 hours (Fig 1A, lower panels). We also generated *DNA2* ^*D245A/-*^ */tet-DNA2* (nuclease-dead) cells from the *DNA2* ^*+/-*^ */tet-DNA2* (S1D Fig). The D245A substitution of chicken Dna2 corresponds to the D294A substitution of human Dna2, which mutation completely abolishes its nuclease activity [24]. We confirmed the expression of *DNA2* ^*D245A*^ mRNA but not of *wild-type DNA2* mRNA at 48 hours after doxycycline treatment (S1E Fig). The suppression of *tet-DNA2* expression caused by doxycycline is hereafter shown as (-). *DNA2* ^*-/-*^ */tet-DNA2*(-) and *DNA2* ^*D245A/-*^ */tet-DNA2* cells stopped proliferating (Fig 1A and 1B), with a significant increase in the sub-G_1_ fraction (S2A Fig, green dots, and S2B Fig).

To investigate the cause of cell mortality, we conducted a chromosomal analysis of mitotic cells. We classified chromosomal aberrations into three categories: exchange, isochromatid-type, and chromatid-type breaks (S2B Fig). The *DNA2* ^*-/-*^ and *DNA2* ^*D245A/-*^ mutants displayed very similar increases in the number of chromosomal aberrations in mitotic chromosome spreads at 72 hours after doxycycline treatment (Fig 1C, 1D and 1E). This phenotype is reminiscent of that seen in cells deficient in HR-related genes such as *MRE11*, *CtIP*, and *RAD51*, suggesting that Dna2 plays an essential role in HR, as do Mre11, CtIP, and Rad51 [12, 13, 25].

**Fig 2:**
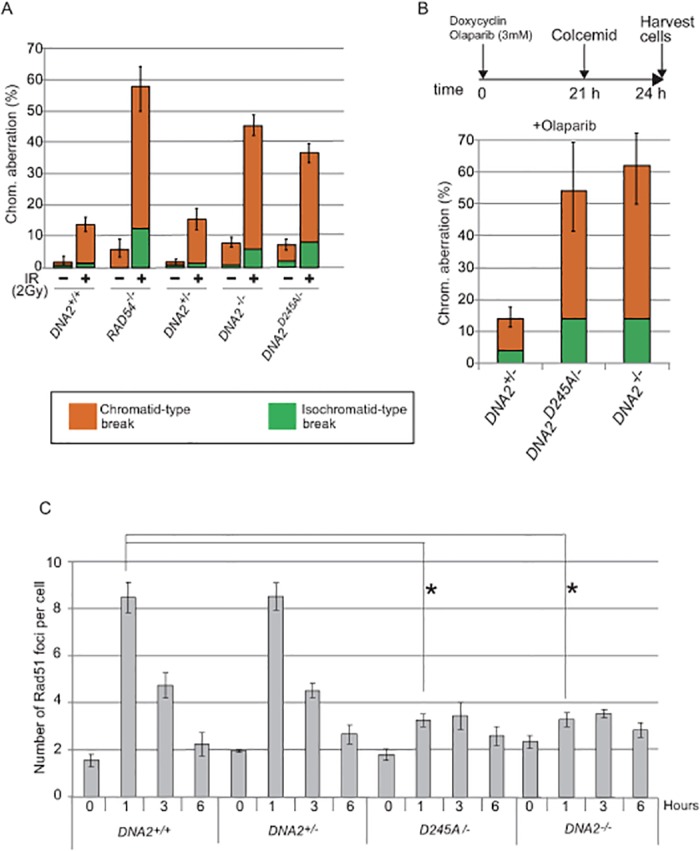
The nuclease activity of Dna2 is required for both HR-dependent DSB repair and Rad51-focus formation. (A) Cells carrying the indicated genotypes were incubated with doxycycline for 24 hours, exposed to 2 Gy γ-rays, and analyzed for chromosomal aberrations in mitotic cells 3 hours after the irradiation. Error bars represent standard deviation calculated from three independent experiments. (B) Top: schematic of chromosome analysis for detecting olaparib-induced breaks. Bottom: Percentage of chromosomal aberrations in mitotic cells carring the indicated genotypes. Error bars represent standard deviation calculated from three independent experiments. At least 100 cells were counted for each cell line. (C) The indicated genotypes were incubated with doxycycline for 24 hours, exposed to IR, and then immunostained with anti-Rad51 antibody at the indicated time post-IR. The average number of Rad51 foci per cell was calculated from three independent experiments. At least 100 cells were counted for each cell line. The representative images are shown in S3A Fig. Error bars were plotted for standard deviation (SD), and *p*-values were calculated using Mann-Whitney U-test. Asterisks indicate a p-value of <0.01.

To examine the role of Dna2 in HR-dependent DSB repair, we exposed the *DNA2* mutants to 2 Gy IR at 24 hours after addition of doxycycline, when the expression of the *tet-DNA2* transgene was undetectable (Fig 1A, lower panels) and Dna2-depleted cells proliferated normally. We then enriched the mitotic cells by adding colcemid, harvesting the cells 3 hours later. This protocol allows for the selective evaluation of the HR efficiency of IR-induced DSBs during the G_2_ phase, where HR predominates in chicken DT40 cells [13, 26]. As with the HR-deficient *RAD54* ^*-/-*^ cells, the number of chromosomal breaks in the two *DNA2* mutants (null and nuclease-dead) was a few times greater than in the *wild-type* cells (Fig 2A), indicating that Dna2 plays an important role in the HR-dependent repair of IR-induced DSBs. Similarly, mitotic chromosomal breaks induced by olaparib, a PARP inhibitor, were four to five times higher in the two *DNA2* mutants than in the *DNA2* ^*+/-*^ cells (Fig 2B). We conclude that the nuclease activity of Dna2 is essential for HR-dependent DSB repair in chicken DT40 cells.

To evaluate Dna2-dependent DSB resection, we monitored IR-induced Rad51-focus formation over time. In *wild-type* and *DNA2* ^*+/-*^ cells, the number of Rad51 foci reached its maximum (8.4 per cell) at one hour after IR (Fig 2C and S3A Fig). The null and nuclease-dead *DNA2* mutants showed a very severe defect in Rad51-focus formation (Fig 2C). Thus we conclude that the nuclease activity of Dna2 plays an essential role in DSB resection in chicken DT40 cells.

### Epistatic Relationship Between CtIP and Dna2 in DSB Resection

**Fig 3:**
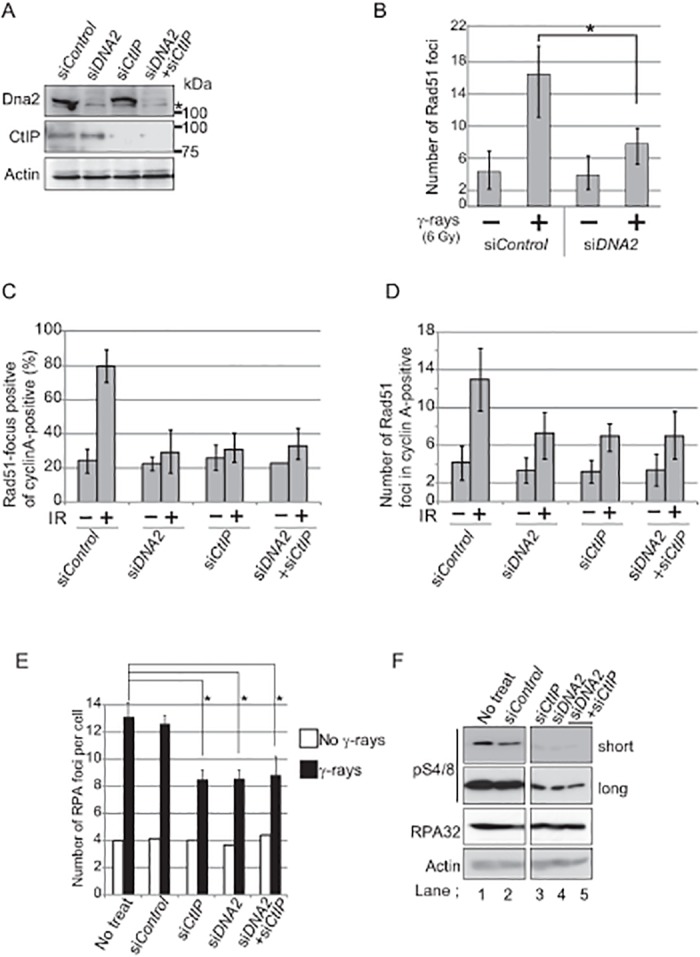
Dna2 and CtIP have an epistatic relationship in both RPA- and Rad51-focus formation. (A) The protein levels of single DNA2- or CtIP-depleted cells and double DNA2/CtIP-depleted cells were analyzed by western blot. Samples were prepared from cells treated for 48 hours with indicated siRNA. An asterisk indicates non-specific staining. (B) Cells treated for 48 hours with siRNA specific for *DNA2* (si*DNA2)* and control siRNA (si*Control)* were exposed to 6 Gy γ-rays, further incubated for 4 hours, and stained with anti-Rad51 antibody. The average number of Rad51 foci per cell from three independent experiments are shown. An asterisk indicates a p-value of <0.001. (C and D) This experimental procedure was the same as in B except that only cells in the S and G_2_ phases (cyclin A-positive cells) were examined. The percentage of cells containing more than three Rad51 foci (C) and the number of Rad51 foci per cell (D) are shown. (E) Number of RPA foci observed 2 hours after irradiation with 6 Gy γ-rays (closed bars) and without irradiation (open bars). Error bars in B, C, D and E indicate standard deviation from three independent experiments. (F) Western blot analysis of cells irradiated with 6 Gy γ-rays was performed using either anti-RPA antibody or anti-phosphorylated RPA (pS4/8) antibody (short and long exposure).

We next performed siRNA-mediated depletion of Dna2 in human U2OS cells (Fig 3A). The depletion of Dna2 resulted in a moderate accumulation of cells in the late S and G_2_ phases (S3B Fig) and caused a significant decrease in the number of IR-induced Rad51 foci (p value < 0.001; Fig 3B), as did the inactivation of Dna2 in chicken DT40 cells (Fig 2C). We obtained very similar results when we examined Rad51-focus formation in cyclin-A-positive S and G_2_-phase cells (Fig 3C and 3D). Likewise, depletion of CtIP in human U2OS cells (Fig 3A) attenuated Rad51-focus formation (Fig 3C and 3D), as did the inactivation of CtIP in chicken DT40 cells [11, 12, 14]. The strong defect in IR-induced Rad51-focus formation in both the CtIP and Dna2 mutants suggests that collaboration between the two resecting enzymes is required for robust DSB resection.

To investigate the functional relationship between CtIP and Dna2, we co-depleted them and subsequently measured Rad51-focus formation. Co-depletion reduced the two proteins to the same degree as did the single depletion of either Dna2 or CtIP (Fig 3A *)*. Remarkably, depletion of both Dna2 and CtIP resulted in a phenotype equivalent to that caused by the depletion of either single gene, in terms of both percentage of Rad51-foci-positive cells and the number of Rad51 foci (Fig 3C and 3D). This implies that the roles of CtIP and Dna2 in DSB resection may be interdependent. To further investigate this interdependent relationship, we examined subnuclear RPA foci as well as the phosphorylation of RPA32 (pS4/8) following IR treatment. Note that the phosphorylation reflects DSB resection [27]. Co-depletion of both CtIP and Dna2 reduced the number of RPA foci to the same extent as did the depletion of either CtIP or Dna2 (Fig 3E and S3D Fig). Likewise, the level of phosphorylated RPA32 was significantly reduced by the depletion of either CtIP or Dna2, whereas co-depletion of both CtIP and Dna2 did not reduce RPA phosphorylation any more than did the single depletion of either CtIP or Dna2 (Fig 3F, compare lanes 3 and 4 with lane 5). We therefore conclude that there may be a mutual dependency between CtIP and Dna2 in DSB resection.

### BRCA1 and CtIP are Required for Recruitment of Dna2 to DNA Damage Sites

**Fig 4:**
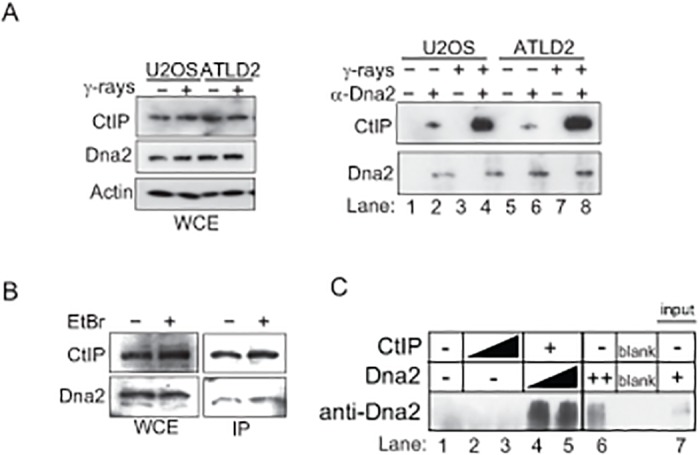
*In vivo* and *in vitro* physical interaction between CtIP and Dna2. (A) Interaction between CtIP and Dna2 is enhanced by IR. U2OS and ATLD2 cells irradiated with 6 Gy γ-rays were subjected to immunoprecipitation with or without anti-Dna2 antibody. The left panel shows proteins included in the whole cell extract (WCE). The right panel shows immunoprecipitated proteins. (B) WCEs were prepared with or without ethidium bromide (final conc. 100 μg/ml) after the treatment of cells with 6 Gy γ-rays, then were subject to immunoprecipition with anti-Dna2 antibody. (C) Interaction between purified CtIP and Dna2. Purified recombinant CtIP (300 ng for lanes 2, 4 and 5, and 600 ng for lane 3) and Dna2 (270 ng for lane 4, and 540 ng for lanes 5 and 6) were incubated and then immunoprecipitated with anti-CtIP antibody. Western blot was conducted using anti-Dna2 antibody. “Input” (lane 7) includes 2.7 ng Dna2.

A simple interpretation of this interdependency is that CtIP and Dna2 function together spatiotemporally at DSB sites. To examine the physical interaction between CtIP and Dna2, we immuno-precipitated the Dna2 protein of whole-cell extract prepared from the human U2OS and Mre11-deficient ATLD2 cell lines [28] (Fig 4A, left panels). In U2OS cells, CtIP protein co-immunoprecipitated with Dna2 even in the presence of ethidium bromide (Fig 4A, right panels, and Fig 4B), suggesting that CtIP does interact with Dna2. Next, we explored the involvement of Mre11 in this interaction, since CtIP physically and functionally interacts with the MRN complex [11]. The extract prepared from Mre11-deficient ATLD2 cell line shows the immuno-precipitation comparable to that from the U2OS cell line (Fig 4A, right panels), suggesting that CtIP forms a robust complex with Dna2 even in the absence of Mre11. This idea is supported by the data that purified recombinant CtIP can physically associate with Dna2 (S2D Fig, and Fig 4C). Importantly, the interaction between CtIP and Dna2 is significantly enhanced by IR (Fig 4A, right panels). We conclude that this interdependency may be based on the physical interaction between CtIP and Dna2.

**Fig 5:**
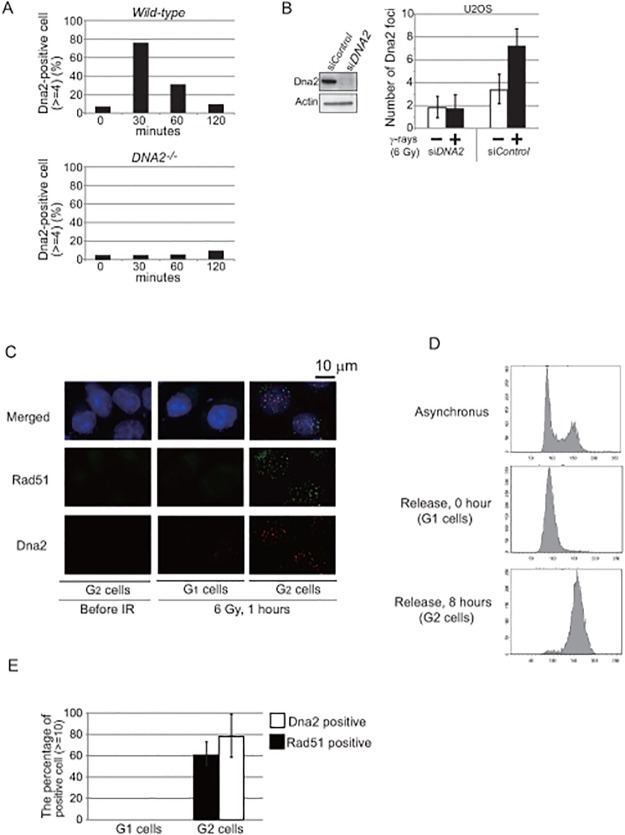
The novel immunostaining allows for the reliable detection of Dna2 localizing at DSB sites. (A) Time-line of Dna2-focus formation following 2 Gy γ-ray irradiation at time zero in *wild-type* and *DNA* ^*-/-*^ chicken DT40 cells. (B) Western blot of Dna2 in human U2OS cells treated with si*DNA2* and si*Control* (left panels) and the average number of Dna2 foci per nucleus in the indicated cells 1 hour after 6 Gy γ-ray irradiation (right histogram). Error bars were plotted for standard deviation. (C) Representative images of IR-induced Dna2 and Rad51 foci in HeLa cells synchronized in the G_1_ and G_2_ phases. (D) Synchronization of HeLa cells using the double thymidine block. Accumulation of cells in G_1_ and G_2_ phase at the time zero and 8 hours, respectively, after releasing cells from the second thymidine block. PI staining is shown on the x-axis (a linear scale) in the histograms. (E) The percentage of γ-ray-induced Dna2 and Rad51 foci positive cells, which have at least 10 foci per nucleus. Error bars were plotted for standard deviation.

This led us to hypothesize that CtIP is required for the recruitment of Dna2 to DSB sites. To test this hypothesis, we examined the recruitment of Dna2 onto IR-induced DSB sites. We checked whether or not any commercially available anti-human-Dna2 antibody could be used to immuno-cytochemically stain Dna2 at DSB sites in γ-ray-irradiated chicken DT40 as well as human cells. We successfully detected Dna2 subnuclear foci in the DT40 cells only after IR with Dna2-focus formation reaching its maximum at 30 minutes and being hardly detectable at 120 minutes after irradiation (Fig 5A and S3C Fig). Dna2 foci were detectable also in γ-irradiated human U2OS and HeLa cells reaching maximal numbers at one hour following irradiation (Fig 5B, and S3E Fig). The absence of discrete subnuclear Dna2 foci in a Dna2-deficient background in both human and chicken cells confirmed the specificity of the immunostaining (Fig 5A, 5B and S3C Fig). IR-induced Dna2 foci were observed only in human cells synchronized in the G_2_ phase, but not in the G_1_ phase (Fig 5C, 5D and 5E). Taken together, we conclude that this novel immunostaining allows for the reliable detection of Dna2 localizing at DSB sites in both chicken and human cells.

**Fig 6:**
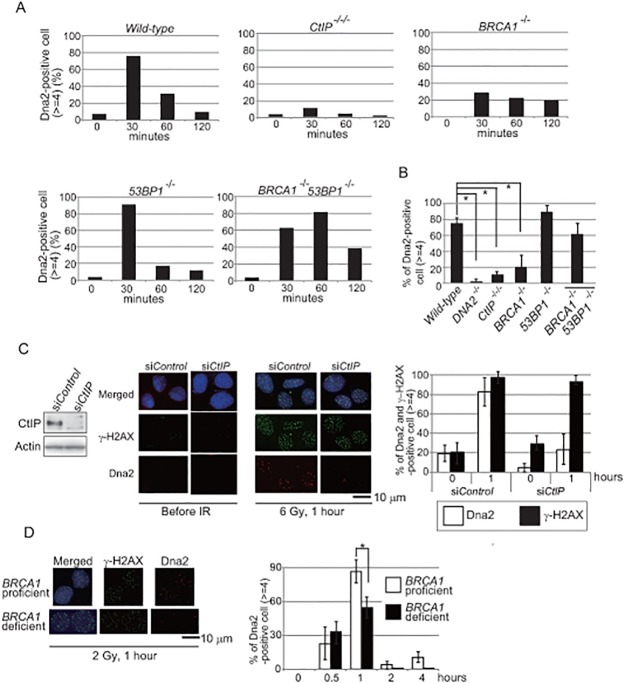
CtIP and BRCA1 are required for the recruitment of Dna2 to DNA damage sites. (A) Time-line of Dna2-focus formation following 2 Gy γ-ray irradiation at time zero in the indicated genotypes of chicken DT40 cells. The y-axis indicates the percentage of nuclei with at least four Dna2 foci. The graph of wild type is the same as in Fig 5A. (B) The percentage of cells with at least four Dna2 foci in chicken DT40 clones carrying the indicated genotypes. Positive cells were counted at 30 minutes following 2 Gy γ-ray irradiation. Asterisks indicate a p-value of <0.01, evaluated by Mann-Whitney U-test. (C) Western blot of CtIP in human U2OS cells treated with si*CtIP* or si*Control* (left panels). Representative images of Dna2 and γ-H2AX foci in human U2OS cells transfected with si*CtIP* or si*Control* for 48 hours (middle images). Percentage of cells displaying at least four Dna2 (open bars) and γ-H2AX (closed bars) foci in the indicated siRNA-treated cells (right histogram). (D) Percentage of cells displaying at least four Dna2 foci in human BRCA1-proficient (MCF7) and-deficient (HCC1937) cells. Error bars in B, C and D indicate standard deviation from three independent experiments.

To test whether CtIP is required to recruit Dna2 to DSB sites, we examined Dna2 foci in CtIP-depleted *CtIP* ^*-/-/-*^ */tet-CtIP* cells when they were still capable of proliferating with normal kinetics. Remarkably, Dna2-focus formation was significantly impaired in the chicken CtIP-depleted cells (Fig 6A, 6B and S3C Fig). Consistently, CtIP depletion by siRNA in human U2OS cells also showed a significant reduction of Dna2 foci (Fig 6C). We therefore conclude that CtIP is required to load Dna2 onto DSB sites. Next, since CtIP physically associates with BRCA1 [29, 30], we tested whether or not BRCA1 is also required to load Dna2 onto DSB sites. Dna2-focus formation was compromised in *BRCA1* ^*-/-*^ DT40 cells (Fig 6A, 6B and S3C Fig) as well as in human cells deficient in BRCA1 (Fig 6D). We analyzed *BRCA1* ^*-/-*^ */53BP1* ^*-/-*^ DT40 cells, since defective Rad51-focus formation in BRCA1-deficient mice is significantly reversed by the additional inactivation of 53BP1 [2, 19]. The *BRCA1* ^*-/-*^ */53BP1* ^*-/-*^ cells showed a higher number of Dna2 foci than did the *BRCA1* ^*-/-*^ cells (Fig 6A, 6B and S3C Fig), indicating that BRCA1 and 53BP1 control DSB resection by regulating the recruitment of Dna2 to DSB sites. In summary, BRCA1 and CtIP are required for the efficient loading of Dna2 onto DSB sites.

Recent studies indicate that CtIP contributes to DSB resection through its non-catalytic role but not as a nuclease [15, 16]. To exclude catalytic role in the recruitment of Dna2 to DSB sites, we generated nuclease-dead *CtIP* mutant cells by inserting N183A and R187A mutations into one of the three allelic *CtIP* gene in DT40 cells [12](S4A–S4C Fig). The resulting *CtIP* ^*N183A*,*R187A/-/-*^ cells were more sensitive to topoisomerase poisons, camptothecin and etoposide, compared with *CtIP* ^*+/-/-*^ cells (S4D and S4E Fig), as indicated previously [15]. As expected, *CtIP* ^*N183A*,*R187A/-/-*^ and *CtIP* ^*+/-/-*^ cells showed very similar Rad51 focus formation whereas CtIP-depleted cells showed virtually no focus formation at one hour after IR (S4G Fig). Thus, the non-catalytic role of CtIP contributes to DSB resection in DT40 cells (S4F Fig). In agreement with this idea, *CtIP* ^*N183A*,*R187A/-/-*^ cells are proficient in Dna2 focus formation (S4H Fig). We therefore conclude that CtIP contributes to the recruitment of Dna2 to DNA damage sites through its non-catalytic role, most likely physical interaction with Dna2, but not as a nuclease.

## Discussion

Our current study shows that the immuno-cytochemical analysis of Dna2 allows for the detection of Dna2 localizing at DNA-damage sites (Fig 5). Dna2-focus formation reached its maximum quickly, at 30 minutes post-IR, and hardly detectable at 120 minutes (Fig 5A). The following evidences suggest that Dna2 foci form at the DSBs that are subjected to Dna2-mediated resection. First, Dna2 foci were detectable only Dna2 proficient cells but not deficient ones (Fig 5A, 5B and S3 Fig). Second, Dna2 foci were undetectable in the G_1_ phase, where no DSB resection occurs (Fig 5C and 5E). Third, the number of Dna2 foci was similar to that of Rad51 foci, and a few times smaller than that of γH2AX foci (Fig 5C, 6C and S3C Fig). Moreover, Dna2 focus formation preceded Rad51 focus formation, which reaches its maximum at 60 minutes post-IR. Forth, prominent Dna2 focus formation was observed only in the cells that are capable of resecting DSBs, including *53BP1* ^*-/-*^ and *BRCA1* ^*-/-*^ */53BP1* ^*-/-*^ cells but not *BRCA1* ^*-/-*^ or *CtIP* ^*-/-*^ cells (Fig 6). We therefore propose that the Dna2 focus formation is an excellent biomarker for ongoing DSB resection. We observed that only few fractions of the IR-induced Dna2 foci colocalize with γH2AX and Rad51 foci (Fig 5C, 6C and S3C Fig). Such differential localization is also often seen between IR-induced Rad51 and γH2AX foci[31]. The differential localization between Dna2 and γH2AX foci may reflect spatial separation of resected DNA from γH2AX in the vicinity of individual DSB sites.

The novel assay of detecting Dna2 foci suggests that CtIP is essential for the recruitment of Dna2 to DNA-damage sites. We also show that CtIP and Dna2 function interdependently in DSB resection (Fig 3C, 3D and 3E). We therefore conclude that CtIP-dependent recruitment of Dna2 is likely required for the DSB resection necessary for efficient HR. By contrast, the functionality of Exo1 appears to be independent of CtIP, since co-depletion of CtIP and Exo1 inhibits DSB resection to a higher extent than that depletion of CtIP alone [32]. Collectively, CtIP is essential for the generation of 3’ overhangs that are long enough to efficiently initiate HR. We thus propose that CtIP and Dna2 function interdependently of one another for the generation of 3’ overhangs that are long enough to efficiently initiate HR.

How does CtIP recruit Dna2 to DNA-damage sites? There are two possible mechanisms underlying the collaboration of the two nucleases. First, CtIP, together with Mre11, might perform short-range resection, with the resulting 3’ single-strand tails efficiently recruiting Dna2, just as the yeast MRX complex stimulates Dna2 resection by creating DNA substrates that efficiently associate with Dna2 [33]. Second, CtIP might facilitate the recruitment of Dna2 to DSB sites via a physical interaction between the two proteins. The latter is more likely since two recent manuscripts [15, 16] demonstrated the catalytic and non-catalytic roles of CtIP endonuclease, with only the non-catalytic role contributing to DSB resection. Our manuscript now defines the non-catalytic role as the recruitment of Dna2 nuclease, the major resecting nuclease to the site of DSBs (S4 Fig). Both CtIP and Dna2 seem to be subject to stringent control by kinases involved in cell-cycle regulation and the damage-checkpoint response [34–36]. The phosphorylation of CtIP and Dna2 might control the interaction between CtIP and Dna2 as well as their enzymatic activity. Moreover, the impaired Dna2-focus formation in *BRCA1* ^*-/-*^ cells but not in *BRCA1* ^*-/-*^ */53BP1* ^*-/-*^ cells (Fig 6B) suggests that 53BP1 and BRCA1 may control CtIP and thereby affect DSB resection by Dna2.

Accumulating evidence points to three distinct functions for BRCA1. First, our current result suggests that BRCA1 contributes to DSB resection by promoting the recruitment of Dna2 to DNA-damage sites (Fig 6A, 6B and 6D). Second, the epistatic relationship between BRCA1 and BRCA2 suggests that the two BRCA proteins collaboratively facilitate polymerization of Rad51 at the resected 3’ overhangs [2]. Third, BRCA1 promotes the elimination of chemical adducts from DSB ends. This third function is suggested by the fact that a mutation at Ser327 in CtIP (equivalent to Ser322 in chicken CtIP) abolishes the tight association of BRCA1 with CtIP and also sensitizes cells to topoisomerase poisons without impairing DSB resection [12, 37]. This third function is also suggested by the finding that RAP80, one of the BRCA1 interacting proteins, contributes to cellular tolerance to the topoisomerase II poison, which generates DSBs covalently associating with polypeptides at their 5’ ends [38]. In summary, BRCA1 contributes to cellular tolerance to various chemotherapeutic agents through three distinct functions.

## Supporting Information

S1 Fig Generation of *DNA2* mutants. (A) Schematic representation of the *DNA2* locus and configuration of gene-disruption with D245A (nuclease-dead) mutation knockin construct. The gene-disruption construct was designed for deletion of exons 6 to 12. The D245A mutation was integrated into exon 5. The boxes represent exons. The relevant *Bam*HI and *Stu*I cleavage sites are indicated. *Bam*HI site (indicated by dotted line with an arrowhead) was integrated with the marker. (B) Southern blot analysis of *wild-type* (*WT*), *DNA2* ^*+/-*^ */tet-DNA2*, and *DNA2* ^*-/-*^ */tet-DNA2* cells using the probe (bold bars) shown in A. Genomic DNA was digested with *Bam*HI and *Stu*I. (C) RT-PCR using the primers shown in A was done 48 hours after addition of doxycycline (Dox). (D and E) Southern blot and RT-PCR were, respectively, carried out for the indicated genotypes. The RT-PCR product amplified from the D245A mutant mRNA, but not *wild-type* mRNA, was digested with *Bst*UI. The silent mutation that generates *Bst*UI site was introduced nearby D245A mutation.
(EPS)

S2 Fig Dna2 nuclease is essential for cellular growth. (A) Cell-cycle distribution of the indicated genotypes. Cells were continuously treated with (+) or without (-) doxycycline (100 ng/ml) for 96 hours. Cells were stained with FITC-conjugated anti-BrdU antibody to measure BrdU incorporation into genomic DNA (y-axis, logarithmic scale) and with propidium iodide (PI) to measure the total DNA (x-axis, linear scale). The large box on the left identifies sub G1 fraction, the lower-middle box identifies G_1_ cells, the upper-middle box identifies S cells, and the lower-right box identifies G_2_ cells. (B) Summary of cell cycle analysis. (C) Representative chromosomal aberrations in chromosome spreads of mitotic cells. Orange, green, and black arrowheads represent chromatid-type, isochromatid-type breaks, and exchanged chromosomes, respectively. (D) Recombinant proteins used in Fig 4C. Dna2 and CtIP were separated by SDS-PAGE and stained with Coomassie Blue. Asterisk marks position of CtIP truncation product.
(EPS)

S3 Fig (A) Representative images of Rad51 foci (green) in the indicated chicken DT40 genotypes at 1 hour after IR. (B) Accumulation of human U2OS cells in late-S and G_2_ phases by knockdown of the Dna2 protein. PI staining is shown on the x-axis (a linear scale) in both histogram and dot plots. The y-axis of dot plots is shown as in S2A Fig. Numbers indicate the percentage of cells in the late-S and G_2_ phases. (C) Representative images merged with Dna2 and γ-H2AX foci of DT40 cells at 30 minutes post-IR. Green, red, and blue indicate γ-H2AX foci, Dna2 foci and DNA, respectively. (D) Representative images of RPA foci in human U2OS cells treated with the indicated si*RNAs*. (E) Kinetics of Dna2-focus formation in both Hela and U2OS cell lines after IR treatment.
(EPS)

S4 Fig Catalytic function of CtIP is required for the tolerance of topoisomerase poisons in chicken DT40 cell. (A) Schematic representation of the chicken DT40 *CtIP* locus and configuration of gene-disruption with N183AR187A (nuclease-dead) mutation knockin construct. The gene-disruption construct was designed for deletion of exons 6 to 10. The N183A, R187A (nuclease-dead) mutation was integrated into exon 7. The boxes represent exons. (B) The flow chart describes the generation process of *CtIP* ^*N183AR187A/-/-*^, *tetCtIP* from *CtIP* ^*+/-/-*^, *tetCtIP cells*. (C) RT-PCR using a couple of primers flanking the mutation region was done 96 hours after addition of doxycycline (Dox). The RT-PCR product amplified from the nuclease-dead mutant (*CtIP* ^*N183AR187A /-/-*^) mRNA, but not *wild-type* mRNA, was digested with *Bfa*I. The silent mutation that generates *Bfa*I site was introduced nearby N183AR187A mutation. (D, E, F) The DT40 nuclease-dead CtIP cells are sensitive to epotoside (D) and campothecin (E), but not to IR (F). The survival curves were plotted using colony formation assays. (G) Representative images of Rad51 foci (green) (upper) and the average number of Rad51 foci per cell in indicated genotypes at one hour after 2 Gy γ-rays (bottom). (H) Representative images of Dna2 foci (Red) (upper) and the percentage of cells with at least four Dna2 foci in chicken DT40 clones carrying the indicated genotypes (bottom). Positive cells were counted at 30 minutes following 2 Gy γ-ray irradiation.
(EPS)
